# Post-Graduate Clinical Charts

**Published:** 1891-06

**Authors:** 


The Post-Graduate Clinical Charts. Designed for use in hospitals and private practice. Arranged and published by Wm. C. Bailey, M. D., and J. H. Linsley, M. D. New York. Copyrighted, 1891, by Drs. Linsley and Bailey.

The need of a clinical chart differing somewhat from any they had seen published induced the authors to prepare these charts.

Both authors are professors in the Post-Graduate Medical School and Hospital, of New York, and their charts were primarily adapted for use in that institution. These charts are unique and give opportunity for very thorough study of cases and very complete records of such study. It is difficult to describe these charts, and, therefore, every one interested should send twenty cents for a sample copy. We may say, however, that these charts are arranged in books of ten each; and a book will give the history of one case for eight weeks.

The first chart records the general characteristics of the patient and has diagrams in outline of chest, front and back, for recording the results of auscultation and percussion. The second chart gives views of the larynx, with ruled spaces for recording the results of laryngoscopic examination and treatment, which results may be graphically represented upon these diagrams. The four succeeding charts give temperature, pulse, and respiration for eight weeks, and the four succeeding charts record all the general symptoms of the patient. Prices are twenty cents for each book, or $2.00 a dozen. Address, Dr. J. H. Linsley, 226 East Twentieth Street, New York. W. M. J.

